# Measuring the Perception of Oder

**Published:** 1891-07

**Authors:** 


### Measuring the Perception of Odor.

At a recent meeting of the French Academy, Secretary Berthelot exhibited a new instrument called the "olfactometer." The inventor is M. Charles Henry. The object of the apparatus is to determine the minimum rate of odoriferous vapor per cubic centimeter of atmospheric air perceptible to the human olfactory nerves. The olfactometer consists principally of a graduated glass tube which moves within a paper envelope. The tube is held to the nose and the paper gradually withdrawn. As soon as the subject of the experiment perceives the odor of the material contained within the glass tube, the latter is withdrawn, and the quantity of vapor which has escaped is calculated from the known capacity of the tube, and the degree marked by the paper envelope. The cubic space affected by the odorous vapor is simultaneusly determined by means of a small areometer. The inventor of the instrument shows that the perceptibility of different odors by different subjects varied enormously, the two limits of his experiments, falling between 2 milligrams of ether per cubic centimetre, and one-thousandth of a milligram of oil of wintergreen per cubic centimetre.

Cocaine Incompatibles.—Cocaine is used in manifold mixtures, and often brought in contact with substances with which it is entirely incompatible. A. Brunner states that it is frequently prescribed with silver nitrate in ointments, when as is probably not known to the prescriber, decomposition of the hydrochloride ensues with formation of insoluble chloride of silver, and a corresponding change in the cocaine. E. Schell, in the Els-lothr *Journ. d. Pharm.*, reports that if calomel and cocaine hydrochlorate are rubbed together chemical reaction sets in. Mercuric oxide, too, if dispensed in form of ointment containing cocaine hydrochlorate, changes so that the ointment instead of producing an anæsthetic effect upon the eyes produces an exceedingly irritating one. This is due to the formation of oxy-chloride of mercury, the quantity of which depends on the amount of cocaine used, the intimacy of its mixture with the oxide, and the age of the ointment.—*Apoth. Ztg.*

